# Disposal of Refuse, Garbage and Sewage in Camps of Instruction1Read at the regular meeting of the Buffalo Sanatory Club, December 14, 1898.

**Published:** 1899-03

**Authors:** Edward Clark

**Affiliations:** Buffalo, N. Y., Ex-Member of the Committee on Disposal of Garbage and Refuse of the American Public Health Association


### DISPOSAL OF REFUSE, GARBAGE AND SEWAGE IN CAMPS OF INSTRUCTION.[1]

By EDWARD CLARK, M. D., Buffalo, N. Y.,
Ex-Member of the Committee on Disposal of Garbage and Refuse of the American Public Health Association.

WHEN people live in thinly populated countries, following as they then do an agricultural or nomadic life, they do not experience to any great degree the evil consequences resulting from the improper and unsanitary methods of the removal and disposal of refuse and excreta of man and animals. It is only when people are thickly gathered together as they are in cities, towns, military camps and garrisons, that the proper disposal of refuse, garbage and sewage becomes a matter of very great importance; it literally becomes a question of life and death and requires the exercise of no small amount of

1 Read at the regular meeting of the Buffalo Sanatory Club, December 14, 1898.

sanitary knowledge and engineering skill to properly solve the problem.

Next to an adequate supply of pure water perhaps no question is of greater importance to a camp or garrison than the sanitary disposition of its refuse and sewage.

Military engineers and medical health officers of the army of today are men of great skill and ability in their particular lines of work, and they have put into use and practice the most modern sanitary rules for the preservation of the health of the troops committed to their care, so that in every well-regulated and properly managed permanent military camp we find very great attention paid to the proper handling of refuse, garbage and sewage.

The great advances made in the last few decades in our knowledge of hygiene and bacteriology has resulted in a great decrease in the death-rate from those diseases that are caused by bad sanitary surroundings, so that we now see a striking contrast in the health of the soldier of today as compared with that of a soldier of a few centuries ago, especially in the middle ages, when no doubt to the barbarous and inefficient methods employed for the removal of the refuse and excreta of man and animals could be traced those frightful ravages of epidemic disease which decimated their ranks.

There is no doubt that even in our own time, many of the fevers of so-called malarial and typhoid character which prevail in military camps are due to a lack of proper attention to this great and all-important subject. Every physician who lays claim to even a small amount of sanitary knowledge, knows that the best authorities of today hold that the specific germ of fevers of the malarial type, "the plasmodium malariæ," is generated by the decomposition of organic material in the soil when the proper amount of heat and moisture are present.

As soldiers in camp suffer much from diseases of this type, it behooves us to lessen their occurrence by preventing a contamination of the soil in and about the camps with organic matter of any kind, and this can only be done by paying strict attention to the sanitary disposal of sewage, garbage and refuse matter of every description.

First, a few words as to the gathering and removal from the camp of refuse and garbage, and under the head of "refuse" I want to include the litter and manure from horses and other animals kept in and about the camp. All this material should be kept in water-tight, covered receptacles, and removed from the camp every morning. The garbage, especially, should be kept in covered cans made of galvanized iron. Before these cans are brought back to the camp they should be thoroughly washed and treated with a disinfectant fluid. All this is necessary to reduce to a minimum the number of flies which are known to be such an important factor in carrying and spreading infectious and contagious diseases.

The place of disposal of refuse and garbage should be at some distance outside of the camp. The wagons or carts in which refuse and garbage are removed from the camps, should have water-tight iron boxes with covers, and after being unloaded they should be washed and thoroughly cleansed before being returned to the camp for further loading.

Various methods can be employed for the ultimate disposal of this garbage and refuse, but I shall speak of all but two of them, only in terms of condemnation. One of the methods which I wish especially to condemn is the practice which I am informed prevailed in some of the camps of mobilisation in this country during the recent war with Spain—that is, allowing the cooks to sell garbage for feeding purposes to irresponsible parties, who would gather it and cart it away from camp in vehicles constructed without any regard to cleanliness or sanitation. This practice cannot be too strongly condemned. This whole business of gathering and disposing of the refuse and garbage in camps should be carried on under the direction of the medical health officer in charge. Another method to be condemned is that of burying the garbage in the immediate vicinity of the camp; and another, that of spreading it upon the surface of the ground near the camp, to be removed when dried. These are dangerous procedures, and no doubt aid greatly in the production of diseases of malarial type, besides vastly increasing the number of flies, which even with the greatest possible precautions are always very numerous in and about a military camp.

For the ultimate disposal of this refuse and garbage only two methods will be considered: First, that of cremation ; second, that of extraction. The first method, that of cremation, implies the total destruction of the refuse by fire. The second, that of extraction, implies a saving of the resultant product, which may be used as a fertilizer.

It is hardly worth while to use the extractive method for the treatment of refuse and garbage of a military camp, for the reason that this method can be economically employed only where there is a large amount of material to be disposed of, as in the case of large cities, since the machinery and apparatus required for the process is very expensive. Furthermore, the best practical results can be obtained by the extractive method only where garbage proper, free from refuse of all kinds, is handled. The garbage and refuse from a military camp would be of a mixed nature, for the destruction of which we must look to cremation as the best possible method.

Different styles of crematories are in use, and I may mention that those of the Mann, Rider & Engel patterns that are in operation in many places, and so far as I can learn, are doing their work satisfactorily. As to time required for construction, it would seem that a crematory could be built in at least thirty days, but, of course, the time required would be influenced somewhat by local conditions, shipment facilities, and the like.

An important question is that which relates to the primary cost of construction. It is impossible to give a definite answer to this query, "for the reason that cost must depend very largely upon availability of materials, command of skilled labor" and the size and capacity of the furnace demanded.

The cost of operation after the furnace is constructed is also of great importance. In estimating this, we must also take into consideration location and available fuel supply. Economical management of the furnace and the class of garbage to be disposed of will also influence the matter to a considerable extent.

The Mann furnace, in Montreal, disposed of miscellaneous refuse, excluding night soil, at 25 cents per ton; this low rate would probably not obtain in a plant on a small scale, such as would be necessary in a camp of instruction. The Engel crematory, in Milwaukee, is said to have disposed of a mixed refuse for 19 cents per ton.

It would seem, however, that the question of cost is not the important one, for "if it shall be determined that the paramount object of the destruction of garbage and refuse is to kill the microörganisms which produce disease, then the expenditure of any amount of money, however great, must not stand in the way of accomplishing this result."

### DISPOSAL OF SEWAGE.

The introduction of an abundant water supply in a military camp is, of course, followed by more or less lavish use of water for domestic purposes, which necessitates a system of sewers for carrying off liquid wastes and human excreta. These sewers must have an outlet, and the proper sanitary disposal of their ejected contents presents for solution a sanitary problem of grave consequences.

The people of the country are awake to the fact that the purity of lakes and streams must be preserved, so that in many states of the Union today, legislative enactments have been secured looking to this much-desired end.

No municipality, village or military camp has any right to allow its wastes to pollute the soil, air or water of any other community. It would be well if an arbitrary law universally prevailed that compelled every community to destroy its own wastes within its own precincts, and not to pass on to its neighbors anything that is contaminated. I am quite certain that I do not exaggerate when I say this end can be accomplished.

There are two methods of sanitary sewage disposal, either of which I think would be eminently fit for adoption and use in a permanent military camp, viz., the method of purification by bacterial oxidation, and the method of chemical treatment, precipitation and sedimentation.

The method of purification by bacterial oxidation was first described and put into practical use by the late Col. George E. Waring. The modus operandi of this process is familiar to all sanitarians, but those who desire to study the subject in detail can procure pamphlets descriptive of the method by addressing the D. A. Tompkins Co., engineers and contractors, of Charlotte, N. C.

I am informed that plants for the treatment of sewage by bacterial oxidation are in successful operation at Willow Grove Park, Pa.; Holmwood Park, Brooklyn; Tuxedo Park, and at the Western House of Refuge for Women, at Albion, N. Y. The plant at the last named place was constructed under the supervision of Messrs. Guthrie & Rockwood, engineers, of this city, at a cost of about $6,000, and I am informed that it will take care of about 50,000 gallons of sewage daily.

From what I can learn of this method, I should consider it a very desirable one for adoption in a permanent military camp. The greatest outlay, of course, would be for primary construction, as, after the plant is completed, the cost for maintenance and labor to operate it is very small.

The treatment of sewage by precipitation and sedimentation has proven a success at Coney Island, N. Y., Round Lake, N. Y., the Soldiers' Home at Bath, N. Y., and at Echota, N. Y. In an article written by Dr. Geo H. Raymond, professor of physiology and sanitary science, at the Long Island College Hospital, he says of this method of sewage disposal: "In presenting to you, therefore, a description of this method of sewage treatment, I am not inviting you to consider a method which exists only in theory, but one which has stood the test of time, and whenever used has given entire satisfaction."

I need not describe the method in detail, as it is familiar to all sanitary officers and engineers who have paid much attention to this subject.

The disinfecting and discharging apparatus is operated automatically by the sewage itself, and it makes no difference whether the tanks are discharged once a week or once an hour. The only objection that can possibly be urged against this method is that the process is considered expensive. It has been demonstrated that the cost of treating chemically 1,000 gallons of sewage is 6 cents; if we allow fifty gallons of sewage a day per capita, which is, I think, a very liberal allowance, it will be seen that the cost for treating the sewage from each individual would be $1.10 per annum, or about one-third of a cent per day; this of course, is the cost of treatment after the plant is completed.

If some of the chemicals used in the process, chlorine, lime, iron, and the like could be recovered, the cost would be very materially reduced, and I think we are safe in saying that in a camp containing from 10,000 to 20,000 troops, the expense for treatment could be reduced to at least 5 cents per 1,000 gallons.

The following claims have been made for this method of sewage treatment, and it would seem that they have all been substantiated:

1. Concentration. The processes of chemical purification for a large town can be carried on in a building 50 x 100 feet or less.

2. Absolute control of all effluvium, which is so thoroughly destroyed by the chemical processes used, that the interior of the building can be kept entirely free from all sewage odors.

3. Economy in operation, the chemical treatment being largely automatic, so that all the labor required would be one man to superintend the works, and the temporary help of two men for one day per week to remove the sludge.

4. Convenience. The sludge can be made innocuous and inoffensive before removal, so that it can be removed in open carts or stored in the neighborhood. It resembles peat in appearance and has no odor.

5. Immunity from infection. The disinfection of the sewage can be carried far enough, thoroughly to kill all pathogenic organisms so that all danger of infection is removed.

